# Beat-to-beat variability of aortic pulse wave velocity: implications for aortic stiffness measurements

**DOI:** 10.1097/HJH.0000000000003935

**Published:** 2025-02-07

**Authors:** Alessandro Giudici, Andrea Grillo, Filippo Scalise, Koen D. Reesink, Tammo Delhaas, Paolo Salvi, Bart Spronck, Gianfranco Parati

**Affiliations:** a Department of Biomedical Engineering, Cardiovascular Research Institute Maastricht (CARIM); b GROW Research Institute for Oncology and Reproduction, Maastricht University, Maastricht, The Netherlands; c Department of Medicine, Surgery and Health Sciences, University of Trieste, Trieste; d Department of Interventional Cardiology, Policlinico di Monza, Monza; e Department of Cardiology, Istituto Auxologico Italiano, IRCCS, Milan, Italy; f Macquarie Medical School, Faculty of Medicine, Health and Human Sciences, Macquarie University, Sydney, Australia; g Department of Medicine and Surgery, University of Milano-Bicocca, Milan, Italy

**Keywords:** arterial stiffness, beat-to-beat variability of pulse wave velocity, invasive aortic pulse wave velocity, validation of pulse wave velocity measuring devices

## Abstract

**Objectives:**

Aortic pulse wave velocity (aPWV) predicts cardiovascular risk. Being the reference method for aortic stiffness evaluation, invasive aPWV is also recommended for validation of noninvasive devices. Because of intrinsic haemodynamic variability and processing issues, aPWV shows beat-to-beat variability. We aimed to quantify this variability and evaluate its implications for the reliability and use of aPWV as reference in validation and clinical application studies.

**Methods:**

The study included *n* = 84 patients, in whom two datasets of invasive data were recorded: 1) simultaneous ascending aorta and iliac pressure acquisitions using a dual-tip intra-aortic catheter, and 2) an additional ascending aorta pressure acquisition. By combining the iliac and ascending aorta pressure recordings from the first and second acquisitions, respectively, we evaluated how a sequential acquisition protocol affects variability. We compared three pressure waveform foot identification methods to investigate the effect of data processing on variability. Furthermore, we estimated how averaging over *n*_beats_ consecutive heartbeats affects the standard deviation (SD) of such *n*_beats_-averaged estimate of aPWV.

**Results:**

The simultaneously acquired invasive aPWV showed a 5% beat-to-beat SD (variability), with small but significant differences between foot identification methods. The sequential acquisition protocol doubled aPWV variability compared to simultaneous acquisition. However, because averaging had a much stronger effect on sequentially measured aPWV, the two acquisition protocols yielded comparable variabilities at *n*_beats_ = 10 (2% vs. 3%).

**Conclusions:**

Our study suggests that, independently from the acquisition protocol and data processing, the intrinsic beat-to-beat variability of aPWV becomes manageable when aPWV values of at least ten heartbeats are averaged.

## Graphical abstract

**Figure:**
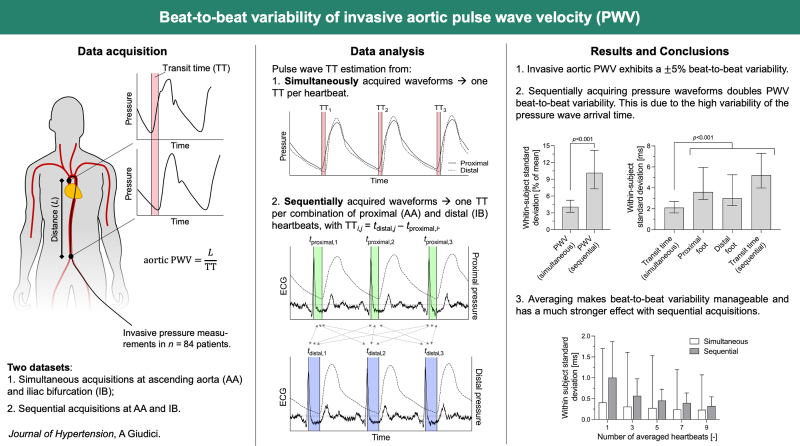

## INTRODUCTION

Aortic pulse wave velocity (aPWV) is a well known cardiovascular risk predictor [1]. In clinical settings, aPWV can be approximated with several noninvasive techniques/devices. Some of these use the transit time (TT)-based measuring principle, consisting of measuring the difference in pulse wave arrival time (i.e., the TT) at two noninvasively accessible arterial sites located in proximity of the aorta's proximal and distal ends [e.g., carotid-femoral PWV (cfPWV)], which yields aPWV given an estimated travelled distance [2–4]. Other techniques indirectly estimate aPWV from the analysis of more peripheral waveforms [3,5]. Despite aPWV's clinical usefulness has been proven with such noninvasive marketed devices, ‘true’ aPWV – i.e., encompassing the entire aorta without involving any other arterial bed – can only be measured by simultaneously positioning pressure catheters at the aortic root and iliac bifurcation. Because highly invasive, this method is superseded by noninvasive techniques/devices in clinical practice. Nevertheless, it remains the most accurate representation of aortic haemodynamics *in vivo* and the reference standard for device validation whenever ‘true’ aPWV is the intended target [6].

Although a patient's PWV is typically defined by a single number, PWV shows beat-to-beat variability *in vivo*. The first and *physiological* source of this phenomenon is the variability of human haemodynamics [7,8]. Because the mechanical behaviour of arteries is highly nonlinear, their stiffness is blood pressure (BP) dependent [9,10]. Hence, any BP fluctuation (including respiration-induced) is mirrored by a contextual PWV change [7,8]. Further, PWV is also heart rate (HR) dependent, likely due to arterial viscoelasticity [11–13]. Furthermore, arterial (including aortic) function can be acutely modulated by the vascular tone, not only altering the artery diameter but also its stiffness and, consequently, PWV [14,15]. Because haemodynamics are constantly regulated to preserve homeostasis, PWV exhibits continuous short-term variability.

A second important variability component stems from the acquisition and processing of the waveforms used to measure aPWV. Concerning data acquisition, the ascending aortic (AA) and iliac bifurcation (IB) pressure waveforms should ideally be acquired simultaneously so that TTs can be calculated on a beat-to-beat basis. However, practically, this is not straightforward and rarely possible. Sequentially, acquiring the two waveforms (e.g., catheter pull-back method) compromises the heartbeat matching, thereby introducing unwanted artificial variability because of the aforementioned beat-to-beat haemodynamic variability. Then, data processing techniques are necessary to extract relevant waveform features (typically, the pressure waveform's foot) for calculating the TT. Different processing methods (currently implemented in noninvasive devices) can be used for foot identification [16–18], and their accuracies may vary depending on inherent assumptions and data quality (e.g., signal-to-noise ratio). These aPWV variability components are *artificial* as they do not reflect actual changes in the arterial wave propagation speed. Nonetheless, together with the *physiological* variability, they affect the accuracy of aPWV measurements, both invasive and noninvasive.

aPWV beat-to-beat variability has direct implications for the validation of aPWV measuring/estimating devices, which requires a reliable reference measurement (preferably invasive aPWV) [6]. A high beat-to-beat variability becomes relevant whenever measurements with the reference and tested devices are not simultaneous or when their measuring/estimating principles differ considerably (e.g., estimation from single vs. multiple heartbeats). Furthermore, aPWV variability also has potential implications for assessing aPWV in clinical practice. Indeed, the reliability of an aPWV estimate is fundamental to detect/monitor its changes over time in a patient. Therefore, recognizing and devising strategies to tackle aPWV variability is pivotal for improving aPWV's clinical utility.

Despite its importance and possible implications for clinical aPWV measurements, literature on the *in vivo* beat-to-beat variability of aPWV (both invasive and noninvasive) is lacking [19]. In this study, we aimed to address this knowledge gap by quantifying the beat-to-beat variability of invasive aPWV, assessing how it is affected by data acquisition and processing, and evaluating its impact for the validation of new devices and for the clinical use of aPWV.

## METHODS

### Study cohort

Details on the study cohort are reported in our previous publication (see Supplementary material) [2]. The study protocol was approved by the Local Ethics Committee of the Monza Policlinic Hospital and followed the principles of the declaration of Helsinki. All participants gave their written informed consent to participate in the study.

### Data acquisition

Aortic pressure waveforms were acquired using a FS-Stiffcath fluid-filled 8Fr double lumen coaxially aligned angiographic catheter (Flag Vascular, Monza, Italy; sampling frequency 977 Hz) designed to record waveforms at two separate arterial sites simultaneously. The distance between the two openings can be adjusted to position each opening at the desired location, with a graduated scale allowing for direct reading of the inter-opening distance. In our study, the catheter was inserted *via* a right femoral artery percutaneous access point, and the central lumen (the catheter's distal opening) was advanced to the AA at 2 cm from the aortic valve under fluoroscopic guidance. The proximal opening was positioned at the IB.

The experimental protocol involved acquiring two datasets: one simultaneous acquisition of AA and IB pressure waveforms and one simultaneous recording of AA and femoral artery pressure waveforms. Electrocardiograms (ECGs) were acquired for both datasets. We discarded the femoral recording from the second dataset and used its AA pressure in combination with the IB pressure from the first dataset to evaluate the effect of using sequential acquisitions of proximal and distal pressures on aPWV variability. The data that support this study are available from the corresponding author upon reasonable request.

### Quantification of the beat-to-beat variability of invasive pulse wave velocity

To evaluate the effect of data processing on aPWV variability, we compared three different identification methods of the pressure waveform's systolic foot: the intersecting tangent (aPWV_IT_), the intersecting interpolant (aPWV_II_), and the second derivative method (aPWV_2ndD_; see Supplementary material). Each method was used to identify the systolic foot time of each heartbeat of both AA (*t*_proximal_) and IB pressure waveforms (*t*_distal_). Their beat-to-beat variability was calculated using the ECG R-wave peak as a time reference, that is, accounting for the summed contribution of the cardiac preejection period and wave propagation time from the aortic valve to the recording site. Then, aPWV calculations followed different methods according to the acquisition modality: simultaneous or sequential.

#### Simultaneous acquisitions

**FIGURE 1:**
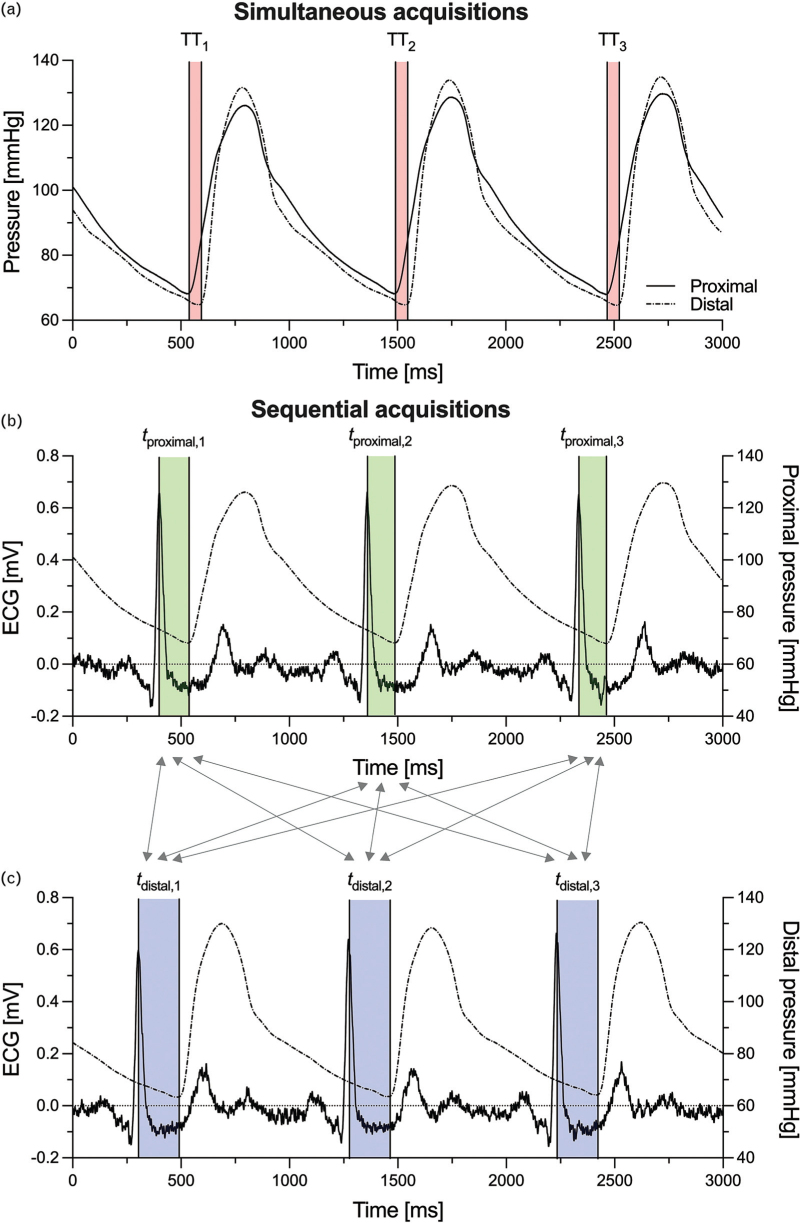
Method of calculation of the transit time (TT) for simultaneously (panel a) and sequentially acquired (panels b and c) proximal and distal pressure waveforms. (Panel a) Because waveforms are acquired simultaneously, each heartbeat's TT can be calculated as the difference between the foot times of the distal and proximal heartbeat waveforms. This yields a number of aortic pulse wave velocity (aPWV) estimates that corresponds to the total number of heartbeats in the recorded signals. (Panels b and c) Because waveforms are acquired sequentially, there is no correspondence between heartbeats in the proximal (panel b) and distal waveforms (panel c). Therefore, a TT value can be obtained for every possible combination of arrival times of the foot of the distal and proximal waveforms, as indicated in this figure's example by nine grey arrows connecting the three *t*_proximal_ values in panel b with the three *t*_distal_ values in panel c. Hence, the total number of aPWV estimates for sequentially acquired pressure waveforms corresponds to the product of total number of heartbeats in the proximal and distal signals (3 × 3 = 9 estimates in this figure's example).

Because of the exact time-matching between heartbeats in the proximal and distal pressure signals, TTs were calculated as heartbeat-specific differences between *t*_distal_ and *t*_proximal_ (Figure 1a). Heartbeat-specific aPWVs were then calculated as the ratio between the inter-catheter opening distance and heartbeat-specific TTs. Hence, each patient's number of aPWV estimates matched the number of heartbeats in the pressure recordings, i.e., on average 56 ± 27 (mean ± standard deviation) heartbeats in our cohort.

#### Sequential acquisitions

Because no matching existed between heartbeats in the two signals, each heartbeat in the proximal pressure was coupled with all those in the distal pressure. Individual TT estimates were, therefore, obtained as the difference between *t*_distal_ and *t*_proximal_ of arbitrarily arranged proximal and distal heartbeats (Figure 1b and c), and the total number of TT and aPWV estimates per patient corresponded to the product between the number of heartbeats in their proximal (on average 51 ± 26 heartbeats) and distal (on average 56 ± 27 heartbeats) pressure waveforms.

### Quantification of the effect of averaging

We evaluated the effect of averaging over *n*_beats_ heartbeats on aPWV variability by averaging aPWV over nonoverlapping blocks of *n*_beats_ consecutive heartbeats and evaluating the variability of these *n*_beats_-averaged aPWV estimates (Figure S1, Supplemental Digital Content). Averages of the means and within-subject standard deviations (SD_ws_) of all the possible combinations of *n*_beats_-averaged aPWV estimates were then considered representative of the patient.

For sequential acquisitions, averaging was performed on the pressure wave arrival times at the AA and IB (i.e., *t*_proximal_ and *t*_distal_, respectively) rather than on aPWV. Then, averaged estimates of TT (and, consequently, aPWV) over *n*_beats_ were calculated for all possible combinations between *n*_beats_-averaged *t*_proximal_ and *t*_distal_ (see the *Quantification of the beat-to-beat variability of invasive pulse wave velocity* section).

Finally, for each patient, we determined a parameter *α* (with 0 ≤ *α* ≤ 0.5) describing the relationship between SD_ws_ of aPWV and the number of averaged heartbeats (see Supplementary material). Notably, the smaller *α*, the stronger the inter-heartbeat dependency and the weaker the effect of averaging on SD_ws_. *α* was calculated for aPWV determined from both simultaneous and sequential acquisitions.

### Statistical analysis

For each individual patient, the beat-to-beat distribution of all variables was evaluated as mean ± SD_ws_. Then, data are presented as either mean ± between-subject SD (SD_bs_) or median [1^st^–3^rd^ quartile] of the within-subject means and SD_ws_ depending on whether they were normally distributed. Overall, all variables but BP and HR were not normally distributed in our cohort.

BP differences between recording sites and BP and HR differences between acquisitions were evaluated using paired Student's *t*-tests. Inter-foot identification method differences of nonnormally distributed variables were evaluated *via* Friedman's tests, followed by Wilcoxon signed-rank tests for pairwise comparisons. Bland–Altman plots were used where appropriate, using 1.96SD_bs_ to identify the 95% confidence intervals. Differences between simultaneous and sequential acquisition protocols were evaluated using Wilcoxon signed-rank tests. Correlation and multivariable linear regression models were used where appropriate. *P* < 0.05 was considered statistically significant.

## RESULTS

### Patient demographics

The study population included ninety-three patients, of which three were excluded for missing acquisitions and six for unusable ECG or pressure waveforms, yielding *n* = 84 analysed patients. Patient characteristics are presented in Table 1. While, as expected, DBP and mean BP were relatively uniform along the aorta, AA systolic BP (SBP) was ∼10 mmHg lower than IB SBP (*P* < 0.001), signifying a change in pressure waveform's morphology between sites. Beat-to-beat pressure variability (both SBP and DBP) was similar at the two recording sites. Furthermore, no difference was found for AA BP and HR between the two datasets.

**TABLE 1:** Patient demographics, blood pressures and heart rate

	Between-subject mean	Within-subject SD
Age (years)	66 ± 11	
Males: females	67 : 17	
**First acquisition**		
Ascending aorta		
SBP (mmHg)	137.5 ± 27.2	4.3 ± 1.7
MBP (mmHg)	95.7 ± 14.2	2.8 ± 1.2
DBP (mmHg)	68.5 ± 10.3	2.2 ± 1.0
Iliac bifurcation		
SBP (mmHg)	147.4 ± 27.3^∗∗∗^	4.3 ± 1.8
MBP (mmHg)	98.8 ± 15.6^∗^	3.0 ± 1.3
DBP (mmHg)	69.4 ± 11.8	2.3 ± 1.0
Heart rate (bpm)	65.1 ± 12.9	2.4 ± 1.9
**Second acquisition**		
Ascending aorta		
SBP (mmHg)	136.8 ± 27.0	4.3 ± 1.8
MBP (mmHg)	94.6 ± 13.9	2.8 ± 1.3
DBP (mmHg)	68.2 ± 9.6	2.2 ± 1.1
Heart rate (bpm)	65.1 ± 13.0	2.6 ± 2.3

Data are reported as mean ± between-subject SD. ^∗^*P* < 0.05 and ^∗∗∗^*P* < 0.001 with respect to ascending aorta. DBP, diastolic blood pressure; MBP, mean blood pressure; SBP, systolic blood pressure; SD, standard deviation.

### Effect of the foot detection algorithm on the beat-to-beat variability

Although different foot identification methods yielded different aPWV estimates (*P* < 0.001, Table 2), they produced similar beat-to-beat variabilities (*P* = 0.112). However, for all methods, aPWV significantly correlated with its SD_ws_ (Figure S2, Supplemental Digital Content). Upon normalisation, aPWV_IT_ and aPWV_2ndD_ variabilities (4.41 [2.92–7.50]% and 5.07 [3.43–6.39]%, respectively) were both higher than aPWV_II_ variability (4.06 [3.11–5.25]%, *P* = 0.005 and *P* = 0.001). Additionally, inter-method correlations of normalized SD_ws_ were, overall, mild (Figure S3, Supplemental Digital Content).

**TABLE 2:** Haemodynamic variables and their beat-to-beat variability

	Intersecting tangent	Intersecting interpolant	2^nd^ derivative	Friedman's test
TT_sim_ (ms)	49.8 [39.4–64.4]^∗∗∗^	49.1 [39.5–66.9]^†††^	52.5 [44.5–67.1]^‡‡‡^	*P* < 0.001
	2.1 [1.4–2.9]^∗^	2.1 [1.6–2.7]	2.4 [1.8–3.4]^‡‡‡^	*P* < 0.001
aPWV_sim_ (m/s)	10.12 [8.08–13.29]^∗∗∗^	10.12 [7.75–12.90]^†††^	9.59 [7.64–11.70]^‡‡‡^	*P* < 0.001
	0.47 [0.27–0.88]	0.41 [0.27–0.63]	0.45 [0.29–0.71]	*P* *=* 0.112
*t*_proximal_ (ms)	131.7 [118.2–148.6]^∗∗∗^	127.3 [111.6–145.2]^†††^	126.5 [115.6–145.4]	*P* < 0.001
	3.9 [2.9–6.5]	3.7 [2.9–6.5]	4.0 [3.1–6.7]	*P* *=* 0.078
*t*_distal_ (ms)	185.4 [167.9–199.1]^∗∗∗^	183.0 [165.2–194.9]^†††^	187.5 [169.3–200.0]^‡‡‡^	*P* < 0.001
	3.1 [2.3–5.3]	3.2 [2.3–5.4]	3.2 [2.4–5.4]	*P* *=* 0.492
TT_seq_ (ms)	49.8 [39.4–63.7]^∗∗∗^	49.3 [39.7–64.8]^†††^	52.6 [44.7–65.8]^‡‡‡^	*P* < 0.001
	5.2 [4.0–7.7]	5.2 [4.0–7.3]	5.4 [4.1–7.9]	*P* *=* 0.770
aPWV_seq_ (m/s)	10.27 [8.16–13.49]^∗∗∗^	10.21 [7.78–13.13]^†††^	9.65 [7.84–11.89]^‡‡‡^	*P* < 0.001
	1.10 [0.63–2.02]^∗∗∗^	1.02 [0.62–1.89]^†^	0.97 [0.58–1.61]^‡‡‡^	*P* < 0.001

For each variable, the top and bottom rows report between-subject median [1^st^–3^rd^ quartile] of the within-subject mean and of the within-subject standard deviation (SD), respectively. ^∗^*P* < 0.05 and ^∗∗∗^*P* < 0.001 with 2^nd^ derivative; ^†^*P* < 0.05, ^††^*P* < 0.01, and ^†††^*P* < 0.001 with intersecting tangent; ^‡‡^*P* < 0.01 and ^‡‡‡^*P* < 0.001 with intersecting interpolant. *t*_distal_, time difference between the electrocardiogram (ECG) R-wave and foot of the iliac bifurcation pressure waveform; *t*_proximal_, time difference between the ECG R-wave and foot of the ascending aortic pressure waveform; TT_seq_ and aPWV_seq_, transit time and pulse wave velocity from sequentially acquired ascending aortic and iliac pressure waveforms; TT_sim_ and aPWV_sim_, transit time and pulse wave velocity from simultaneously acquired ascending aortic and iliac pressure waveforms.

aPWV differences reflected those in TT (Table 2), which, in turn, arose from inter-method discrepancies in the identification of *t*_proximal_ and *t*_distal_. Interestingly, while *t*_proximal_ and *t*_distal_ showed comparable between-method biases, related confidence intervals were 3–5-fold larger for *t*_proximal_ than for *t*_distal_ (Figure S4, Supplemental Digital Content). This finding, together with the higher SD_ws_ of *t*_proximal_ (Table 2, *P* < 0.001 for all methods), indicates a lower consistency in the processing of the proximal pressure waveform by all methods.

### Comparison between simultaneous and sequential acquisition protocols

Sequentially acquiring pressure waveforms increased TT's SD_ws_ by more than twofold (Table 2). This increased variability was mainly due to the high variability of *t*_proximal_ and *t*_distal_, which was 1-to-2-fold higher than TT variability from simultaneous acquisitions (Table 2, *P* < 0.001 for both *t*_proximal_ and *t*_distal_ and for all methods) and led to a twofold increase in aPWV variability (*P* < 0.001). Notably, despite unchanged TTs, the sequential acquisition protocol yielded significantly higher aPWV values (*P* < 0.001 for all methods). Indeed, mathematically, given TT distributions with the same mean but different SDs, the distribution with the highest variability always yields the highest mean PWV.

### Effect of averaging on the variability of invasive pulse wave velocity

**FIGURE 2:**
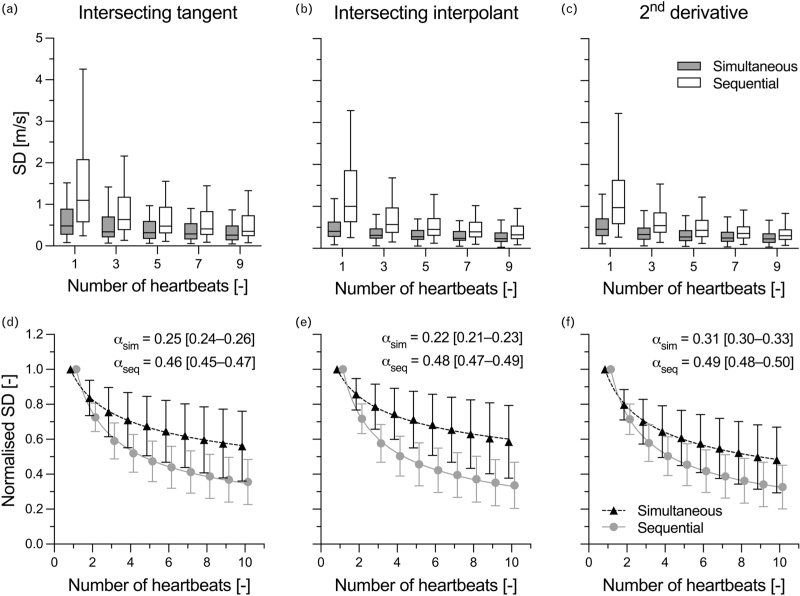
Effect of averaging on the beat-to-beat variability of invasive aortic pulse wave velocity (aPWV). Averaging reduces the variability of aPWV from sequential pressure acquisitions at a much faster rate than that of aPWV from simultaneous acquisitions. (Panels a–c) The effect of averaging is shown in terms of median within-subject standard deviation (note that outliers are not shown to improve figure readability. See Supplementary material, Figure S5 for complete graphs). (Panels d–f) The within-subject standard deviation of the *n*_beats_-averaged aPWV is normalized with respect to that of the nonaveraged aPWV (i.e., *n*_beats_ = 1) to allow for an easier estimation of *α*, which is shown as best fit value [95% confidence interval].

Averaging over consecutive heartbeats reduced aPWV variability for both acquisition protocols (Figure 2). However, while for sequential acquisitions SD_ws_'s dependency on *n*_beats_ approximated closely that of a stochastic distribution (*α* ≅ 0.50), this rate was much reduced for simultaneous acquisitions (*α* ≅ 0.25, Figure 2d–f), signifying a strong inter-dependency between consecutive heartbeats. Averaging over *n*_beats_ = 7 [3–15] (aPWV_IT_), 5 [3–10] (aPWV_II_), and 6 [3–11] (aPWV_2ndD_) was necessary to achieve a certainty on aPWV's mean from sequential acquisitions which equalled that of nonaveraged aPWV from simultaneous acquisitions. With simultaneous acquisitions, averaging over *n*_beats_ = 10 heartbeats as indicated in the device validation recommendations [6] reduced aPWV variability to 0.23 [0.12–0.50] (aPWV_IT_), 0.22 [0.15–0.38] (aPWV_II_), and 0.22 [0.12–0.36] m/s (aPWV_2ndD_). Note that, although a sequential acquisition design required *n*_beats_ = 26 [12–72], 26 [11–72] and 17 [7–48] to equal these levels of variability, variability at *n*_beats_ = 10 was already comparable to that attained with simultaneously acquired waveforms (0.34 [0.24–0.70], 0.29 [0.19–0.42], and 0.30 [0.20–0.53] m/s, respectively).

### Relationship between pulse wave velocity, blood pressure and heart rate variability

**FIGURE 3:**
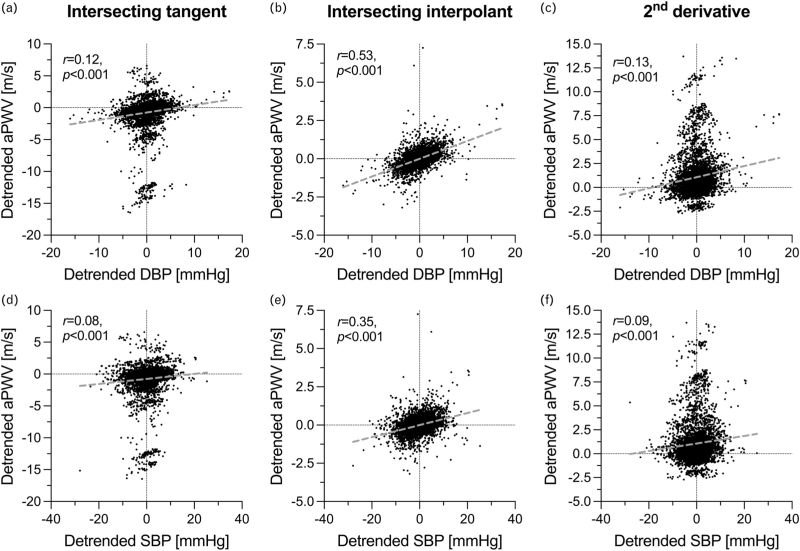
Relationship between blood pressure and invasive aortic pulse wave velocity (aPWV). Each datapoint represents a single heartbeat from a patient of our cohort. Both aPWV and blood pressure are detrended (i.e., the mean over the entire acquisition is subtracted from individual heartbeat values) to aid comparability between subjects. Nondetrended plots are presented in Supplementary material, Figure S6. Note that for aPWV_IT_ and aPWV_2ndD_, single heartbeat aPWV readings significantly deviated from the within-subject mean (panels a, c, d, and f), compromising the relationship with blood pressure.

The variability of aPWV_IT_ and aPWV_2ndD_ did not correlate with either HR or BP variability (except AA DBP for aPWV_2ndD_, *r* = 0.24, *P* = 0.026). This was likely due to both methods failing to accurately estimate aPWV over some heartbeats (Figure 3a, c, d, and f), which compromised the relationship with BP. Conversely, aPWV_II_ significantly correlated with all pressures (AA DBP: *r* = 0.29, *P* = 0.006 and SBP: *r* = 0.32, *P* = 0.003; IB DBP: *r* = 0.36, *P* = 0.001 and SBP: *r* = 0.32, *P* = 0.003), suggesting that this method may be more robust than the other two. We also evaluated the relationship between aPWV and BP on an individual heartbeat basis across the entire cohort, detrending both variables by subtracting their mean to aid between-subject comparability (Figure 3). Only aPWV_II_ yielded a clear relationship with BP (Figure 3b and e), although statistically significant correlations were found for all three methods. Furthermore, DBP more strongly correlated with aPWV than SBP (Figure 3a–c vs. d–f). Indeed, in a multilinear regression model with DBP, PP, and HR as predictors, beat-to-beat aPWV_II_ changes were significantly associated with those in DBP and HR (*β* = 0.098 m/(s mmHg) and 0.012 m/(s bpm), respectively, *P* < 0.001 for both), but not with changes in PP.

## DISCUSSION

### Main findings

The beat-to-beat variability of human haemodynamics is a long known and well studied phenomenon [7,8]. Nonetheless, literature on PWV beat-to-beat variability is extremely sparse, despite its potentially important implications for clinical practice. Here, we addressed this knowledge gap by analysing the beat-to-beat variability of invasive aPWV, given its importance as reference standard for noninvasive aPWV measurement. Our major findings were: 1) in the most ideal measuring conditions (i.e., simultaneous invasive acquisitions), aPWV shows a beat-to-beat variability of approximately 5% of its mean, 2) data processing (e.g., different foot identification methods) accounts for a considerable part of this variability, 3) the high variability of the pressure waveform arrival time (i.e., *t*_proximal_ and *t*_distal_) dramatically increases aPWV variability when pressure waveforms are acquired sequentially, and 4) because of the inter-relationship between consecutive heartbeats, averaging more effectively reduces variability for sequential than simultaneous acquisition protocols.

### Physiological explanation of beat-to-beat variability

The existence of beat-to-beat aPWV variability *in vivo* is unsurprising, given aPWV's acute dependency on BP, HR, and vascular tone [10–12,14]. Nonetheless, in this study, patients’ haemodynamics were relatively stable. Indeed, acquisitions were relatively short and performed at rest, suggesting that vascular tone changes unlikely played a major role in the observed aPWV variability. The average DBP beat-to-beat variability was just above 2 mmHg. Given aPWV estimation from the pressure waveform foot, DBP is the most suitable determinant of its pressure dependency (confirmed here by its strongest relationship with aPWV) [20,21]. By a rule of thumb, a 10 mmHg increase in DBP yields a ∼1 m/s increase in cfPWV [21], which scales to ∼0.2 m/s for the DBP variability in this study. Assuming that arterial BP and diameter relate exponentially leads to similar estimations [9]. Our data also indicated a similar dependency, especially for aPWV_II_ (Figure 3b, linear regression slope: 0.12 m/s per mmHg). Additionally, HR beat-to-beat variability was also small (∼2 bpm). Previous studies showed that HR's isolated impact on PWV is one order of magnitude smaller than that of BP [11,13], which is in line with the results of our multilinear regression model. This suggests that HR variability did not majorly contribute to the observed aPWV variability. Hence, overall, haemodynamic variability was likely responsible for only half of the ∼0.4 m/s aPWV variability found herein, which suggests that a comparable variability component was artificial in nature.

### Effect of different foot detection methods

Similar to previous work [16,18,22], we found significant aPWV differences between foot detection methods. Median aPWV_2ndD_ was ∼0.7 m/s lower than both aPWV_IT_ and aPWV_II_, which clinically translates to a 7–11% estimated difference in cardiovascular risk [1,23] and underlines the importance of consistently using a single foot detection method in patient follow-up. Inter-method differences in beat-to-beat variability were milder, which agrees with previous work on the variability of repeated PWV readings [24]. Nonetheless, the intersecting interpolant method showed a slightly but significantly lower variability than both other methods. This possibly explains why only aPWV_II_ showed a clear relationship with BP: the lower accuracy of the other two methods likely partially masked such relationship (see outliers in Figure 3a, c, d, and f). Furthermore, different methods agreed poorly at an individual patient level, i.e., in discerning patients with high/low variability (Figure S3, Supplemental Digital Content). To further investigate inter-method discrepancies, we compared their identification of the foot of the AA and IB pressure waveforms (Figure S4, Supplemental Digital Content) and found a much poorer inter-method agreement at the AA. Indeed, in our data, the AA upstroke appeared more variable in shape and slope than the IB upstroke (Figure S7, Supplemental Digital Content), possibly due to the recording site's proximity to the heart or to the overlapping of reflected waves originating from potentially any district of the arterial tree. Because of this physiological variability in AA pressure morphology, its processing constituted a principal source of artificial variability in our study. Unlike the 2^nd^ derivative and intersecting tangent methods, the intersecting interpolant method relies on the slope of an entire portion of the pressure upstroke (5–50% in this study) to determine the foot, which may explain its higher robustness against waveform variability.

### Effect of different acquisition protocols

Sequentially acquiring AA and IB pressures constituted the major source of artificial aPWV variability, more than doubling within-subject SDs compared to simultaneous acquisitions. This observation may have important implications for translation in noninvasive measurement settings. Currently, both measurement protocols are common in marketed devices, with no agreement on the superiority of one method over the other. The observed additional variability in sequential acquisitions may reflect the high beat-to-beat variability of cardiac and not vascular function. Specifically, the variability of the duration of the preejection period (comparable to that reported in previous work [25]) was much higher than that of the wave propagation time (TT) along the aorta. Our findings confirm those of Zhang *et al.*[26], who reported that the BP-induced beat-to-beat variability of the pulse arrival time (i.e., the sum of the preejection period and TT) was higher than that of TT in dogs. The preejection period variability only affects the TT estimation with a sequential acquisition protocol, when each arbitrarily arranged pair of proximal and distal heartbeats is characterized by two distinct preejection times, the variability of which will be reflected in that of aPWV.

### Implications for device validation

The beat-to-beat variability of invasive aPWV has direct implications for the validation of new noninvasive aPWV devices. Current device validation recommendations consider a three-tier system, with new devices classified as either *good*, *acceptable*, or *poor (failed)* according to the bias and the SD of this bias with respect to the reference invasive aPWV measurement [6]. In line with guidelines for validating BP devices [27], this bias and SD are combined to calculate the error threshold within which 85% of the device comparisons fall. Note that, since both the bias and its SD are considered, a device under test with a relatively large bias must have a relatively small SD to pass validation. While our data cannot provide any information on the bias itself, its SD arises from the SD of aPWVs obtained with both the tested device and the reference invasive method. Hence, invasive aPWV's variability reported herein directly impacts the validation of noninvasive devices (i.e., the higher the variability of the reference, the harder for a noninvasive device to pass validation). Our findings may suggest that using a sequential acquisition protocol strongly reduces the chances of a tested device to pass validation via two effects. First, a sequential acquisition design more than doubles aPWV beat-to-beat variability. However, our data show that the effect of the acquisition protocol becomes marginal once averaging over ten heartbeats, as indicated in the recommendations, is performed (SD: 0.22 vs. 0.29 m/s, yielding a ∼0.00–0.05 m/s increase in the SD of the bias over three repeated measurements). Indeed, the sequential acquisition protocol effectively neutralizes the interdependency between consecutive heartbeats. As a result, averaging has an approximately twice as strong effect on sequentially than simultaneously acquired aPWV. This observation is pivotal for a widespread adoption of noninvasive PWV measurements, given that a sequential acquisition protocol is deemed to be more applicable in daily clinical practice. Second, an increased TT variability introduces a small but systematic positive bias on the aPWV mean (here ∼0.1 m/s, aPWV_sim_ vs. aPWV_seq_ in Table 2). These two effects combined increase the error threshold by 0.10–0.15 m/s when using a sequential compared to a simultaneous acquisition protocol, thereby slightly worsening chances of a tested device to pass validation.

### Limitations

Here, we compared three different foot identification methods. While other methods have been proposed previously [16,22], these three methods are those most frequently used in clinical studies and implemented in marketed devices. We conducted a broader comparison on a subset of our cohort and found that other methods yielded comparable aPWV variabilities. Hence, our results do not lose generalizability.

We evaluated the beat-to-beat variability of invasive aPWV. While invasive aPWV is the reference standard measurement, it is not applicable in everyday clinical practice, where noninvasive aPWV measurements are indeed more common. Because noninvasively acquired arterial waveforms (e.g., ultrasound, tonometry) likely have a lower signal-to-noise ratio than invasive catheter-derived pressure, our results cannot be directly extended to noninvasive aPWV. Replicating our analysis on the beat-to-beat variability with other metrics, e.g., cfPWV, would be useful in future investigations. Nonetheless, by providing data on PWV variability measured under near-ideal conditions (invasive simultaneous acquisitions), our findings will be useful for interpreting those future investigations. Additionally, we provided insights into how data acquisition (i.e., simultaneous vs. sequential) and processing (i.e., foot identification method) affect aPWV variability, which remain relevant also for noninvasive acquisitions.

## CONCLUSION

aPWV shows both physiological (due to haemodynamic variability) and artificial (due to data acquisition and processing) beat-to-beat variability. This variability does not represent an issue for the validation of noninvasive aPWV devices whenever appropriate averaging aPWV values of at least ten heartbeats is performed, regardless of data acquisition and processing choices.

## ACKNOWLEDGEMENTS

None.

### Conflicts of interest

The authors have no conflict of interest to disclose.

## Supplementary Material

Supplemental Digital Content
