# Supplementary material for: Beat-to-beat variability of aortic pulse wave velocity: implications for aortic stiffness measurements
Source: J Hypertens. 2025 Feb 7;43(4):589–97. doi: 10.1097/HJH.0000000000003935 (PMC11872263; doi:10.1097/HJH.0000000000003935)
Supplement: Supplemental Digital Content [file jhype-43-589-s001.pdf]

Beat-to-beat variability of invasive aortic pulse wave velocity: implications for aortic stiffness measurements

Alessandro Giudici, Andrea Grillo, Filippo Scalise, Koen D Reesink, Tammo Delhaas, Paolo Salvi, Bart Spronck*, Gianfranco Parati*

Supplementary material

*These authors contributed equally to this work.

Correspondence to Bart Spronck, PhD,

Department of Biomedical Engineering,
Cardiovascular Research Institute Maastricht (CARIM),
Maastricht University, PO Box 616,
6200 MD Maastricht, The Netherlands.

E-mail: b.spronck@maastrichtuniversity.nl

S1. Details on the study cohort

The study cohort consisted of all suitable consecutive patients undergoing angiography at the Interventional Cardiology Unit of Monza's Polyclinic Hospital (Monza, Italy) over a 2-month period. Exclusion criteria were age <18 years, body mass index >35 kg/m^2, emergency hospitalisation, heart failure with unstable haemodynamic conditions, atrial fibrillation or paced cardiac rhythm, low ejection fraction, severe valvular disorders, and known significant stenosis of the carotid and/or femoral artery.

S2. Foot detection methods and data filtering

The intersecting tangent method identifies the point of the systolic upstroke with the highest pressure–time slope (i.e., the zero-crossing in the second derivative of the pressure upstroke). Then, the tangent line to the pressure waveform at this point is intersected with the horizontal line passing by the end-diastolic pressure minimum to yield the time of the waveform foot.[16] The intersecting interpolant method follows a similar principle but uses the interpolating line of an entire portion of the pressure upstroke instead of the tangent to a single point with the highest slope.[2] In this work, the interpolating line was determined over the portion of the pressure upstroke that spans between 5% to 50% of the pulse pressure (PP). Finally, the second derivative method identifies the waveform foot as the point which presents the fastest increase in slope over time.[16] This point can be identified *via* the highest peak of the second derivative of the pressure upstroke.

For each patient, proximal and distal pressure waveforms were first filtered using the phase-preserving 2nd order Savitzky-Golay filter with a window of 31 sampling points (i.e., ~31.7 ms) to increase the signal-to-noise ratio. Then the three foot identification methods were used to identify the systolic foot of each heartbeat of both the ascending aortic and iliac bifurcation pressure waveforms. Note also that because derivatives act like high-pass filters (i.e., decrease the signal-to-noise ratio), a second 2nd order Savitzky-Golay filter with a window of 51 sampling points (i.e., ~52.2 ms) was applied to the second derivative of the pressure signals to allow for the identification of their peak.

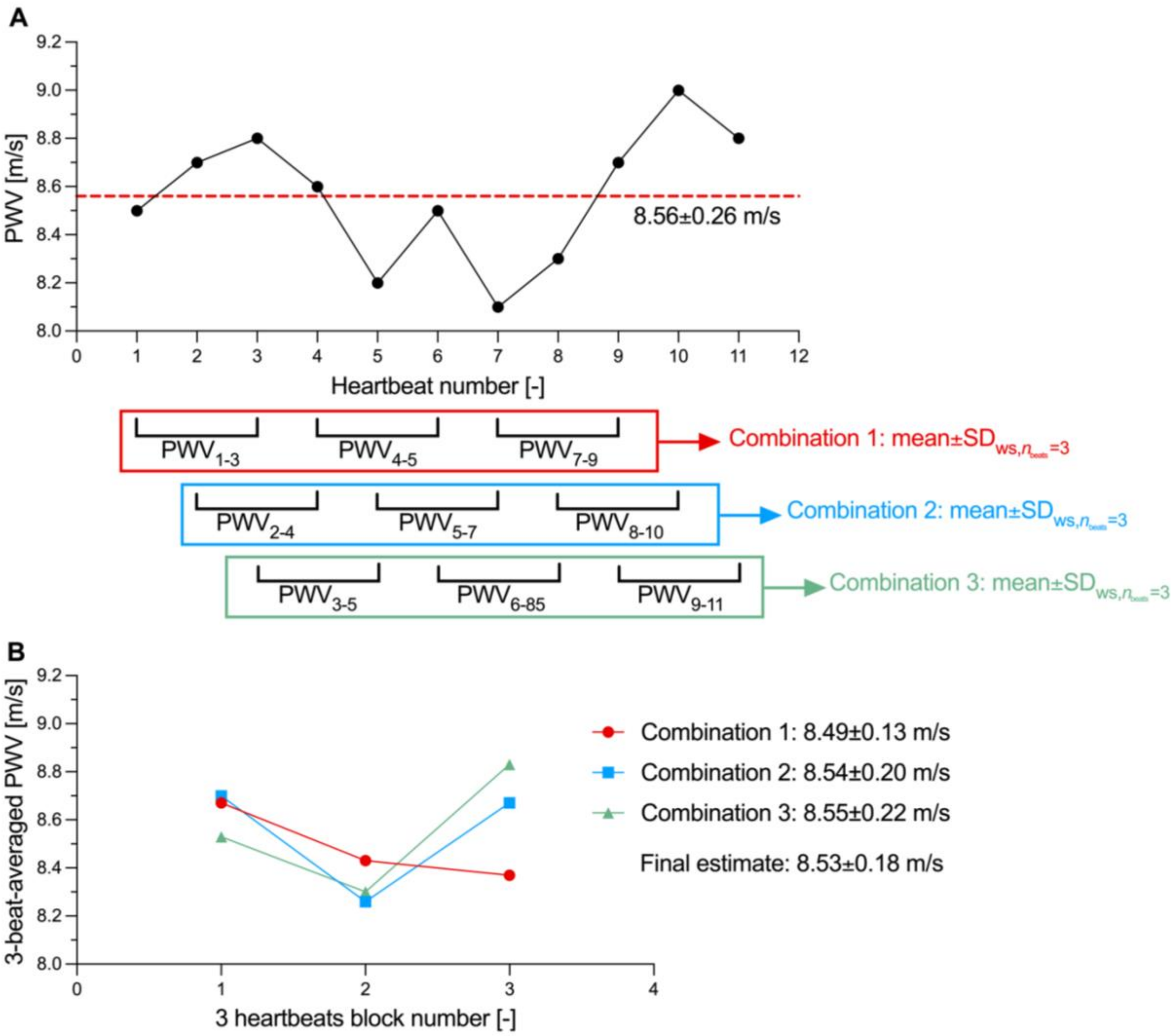

Figure S1 – Method for the estimation of the effect of averaging of n_{beats} consecutive heartbeats (in this example, $n_{\text{beats}} = 3$). All possible combinations of consecutive blocks of n_{beats} consecutive heartbeats are identified. For each combination, the mean and standard deviation (SD) of the n_{beats}-averaged PWV are calculated. The average of the mean and SD of all combinations are considered representative of the patient.

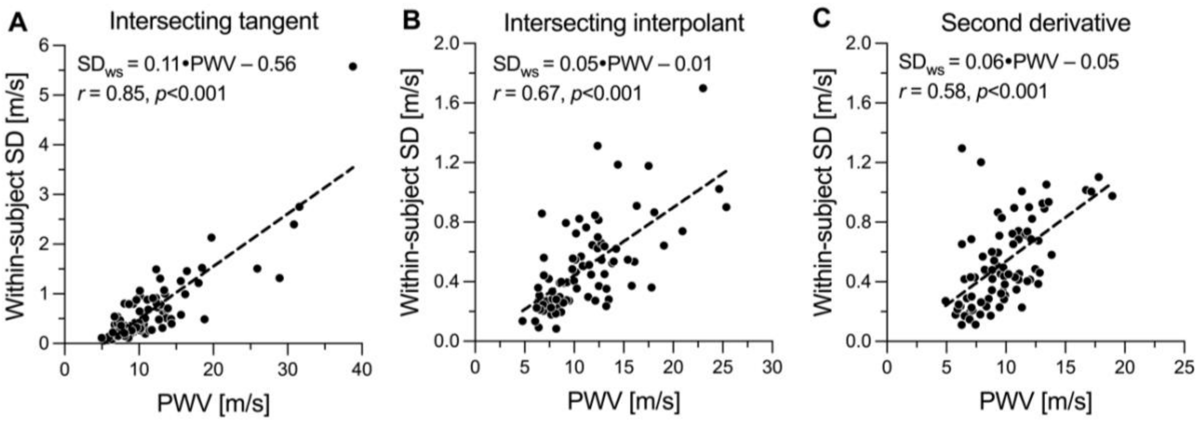

Figure S2 – Correlation between within-subject standard deviation (SD_{ws}) and mean of invasive PWV as estimated with the intersecting tangent (Panel A), intersecting interpolant (Panel B) and second derivative (Panel C) methods.

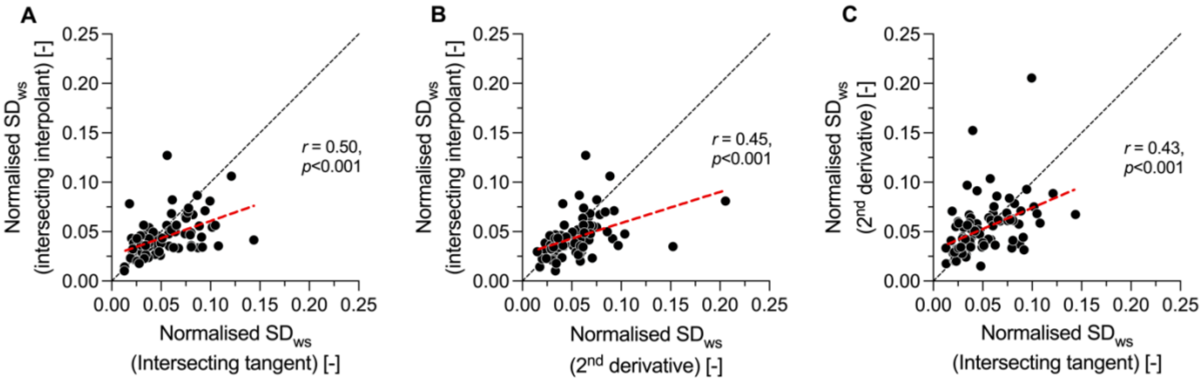

Figure S3 – Correlation between normalised within-subject standard deviations (SD_{ws}) of invasive aortic PWV determined using three pressure waveform foot identification methods. Panel A: Intersecting tangent vs intersecting interpolant; Panel B: 2nd derivative vs intersecting interpolant; Panel C: intersecting tangent vs 2nd derivative. Normalised SD_{ws} was calculated as the ratio between SD_{ws} and the mean of PWV.

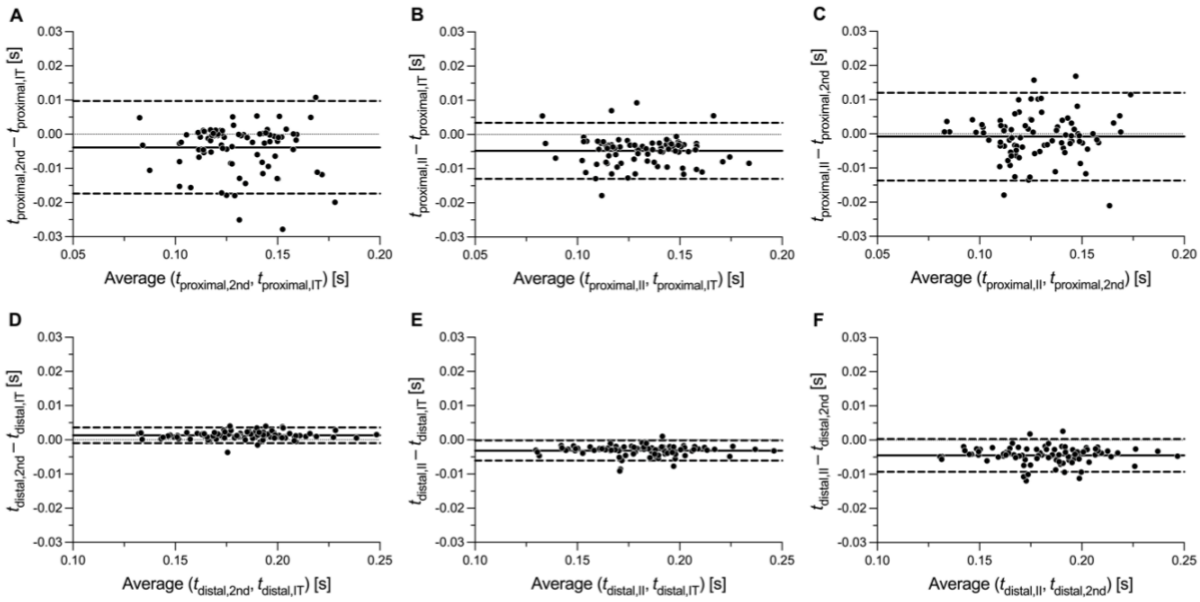

Figure S4 – Agreement on the identification of the proximal (t_{proximal}, i.e., at the ascending aorta) and distal pressure wave foot (t_{distal}, i.e., at the iliac bifurcation) between the intersecting tangent (IT), 2nd derivative (2nd) and intersecting interpolant (II) methods. Panels A and D present the Bland-Altman comparison between the IT and 2nd methods for t_{proximal} and t_{distal}, respectively. Panels B and E present the Bland-Altman comparison between the IT and II methods for t_{proximal} and t_{distal}, respectively. Panels C and F present the Bland-Altman comparison between the II and 2nd methods for t_{proximal} and t_{distal}, respectively.

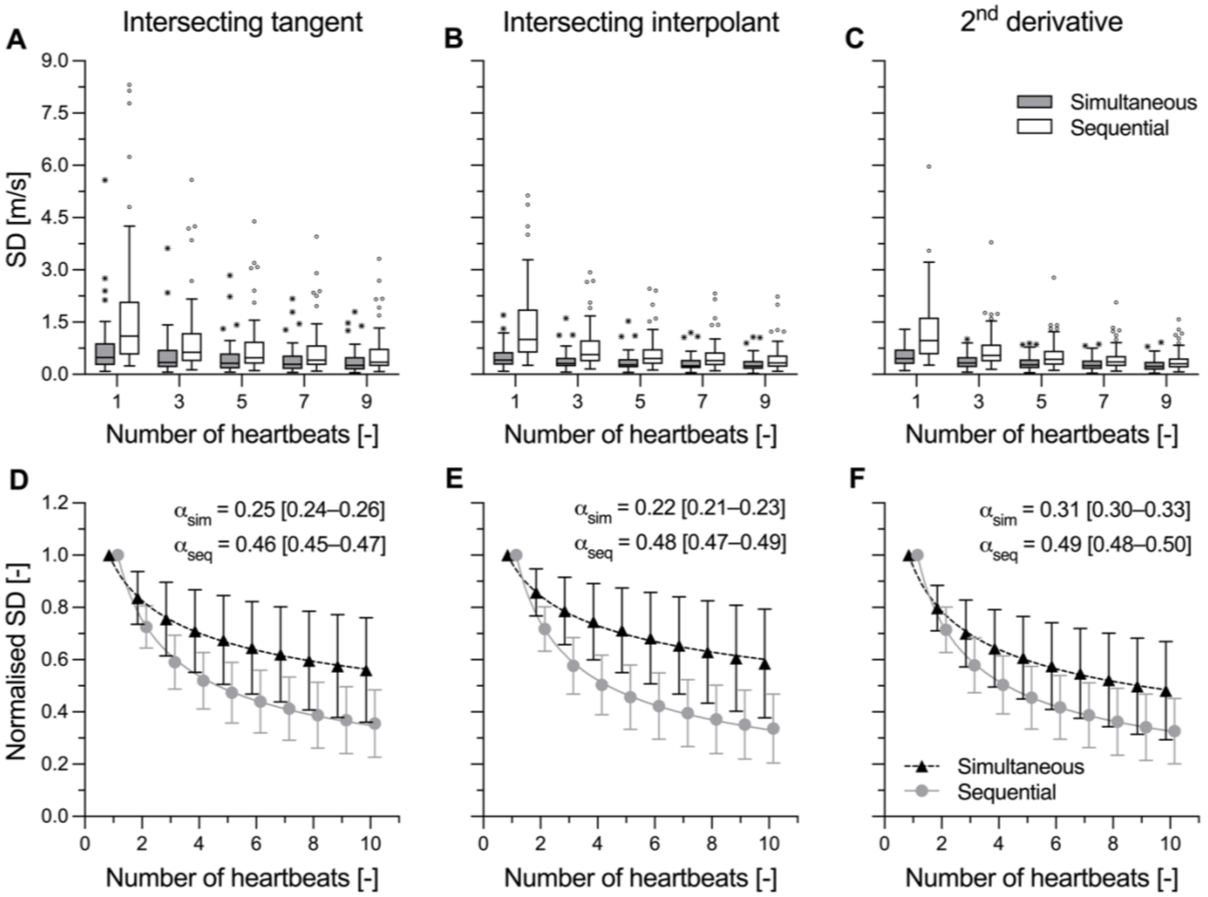

Figure S5 – Effect of averaging on the beat-to-beat variability of invasive ascending aorta to iliac bifurcation pulse wave velocity (PWV): Averaging reduces the variability of PWV from sequential pressure acquisitions at a much faster rate than that of PWV from simultaneous acquisitions. Panels A–C: The effect of averaging is shown in terms of median within-subject standard deviation. Panels D–F: The within-subject standard deviation of the n_{beats}-averaged PWV is normalised with respect to that of the non-averaged PWV (i.e., n_{beats} = 1) to allow for an easier estimation of α, which is shown as best fit value [95% confidence interval].

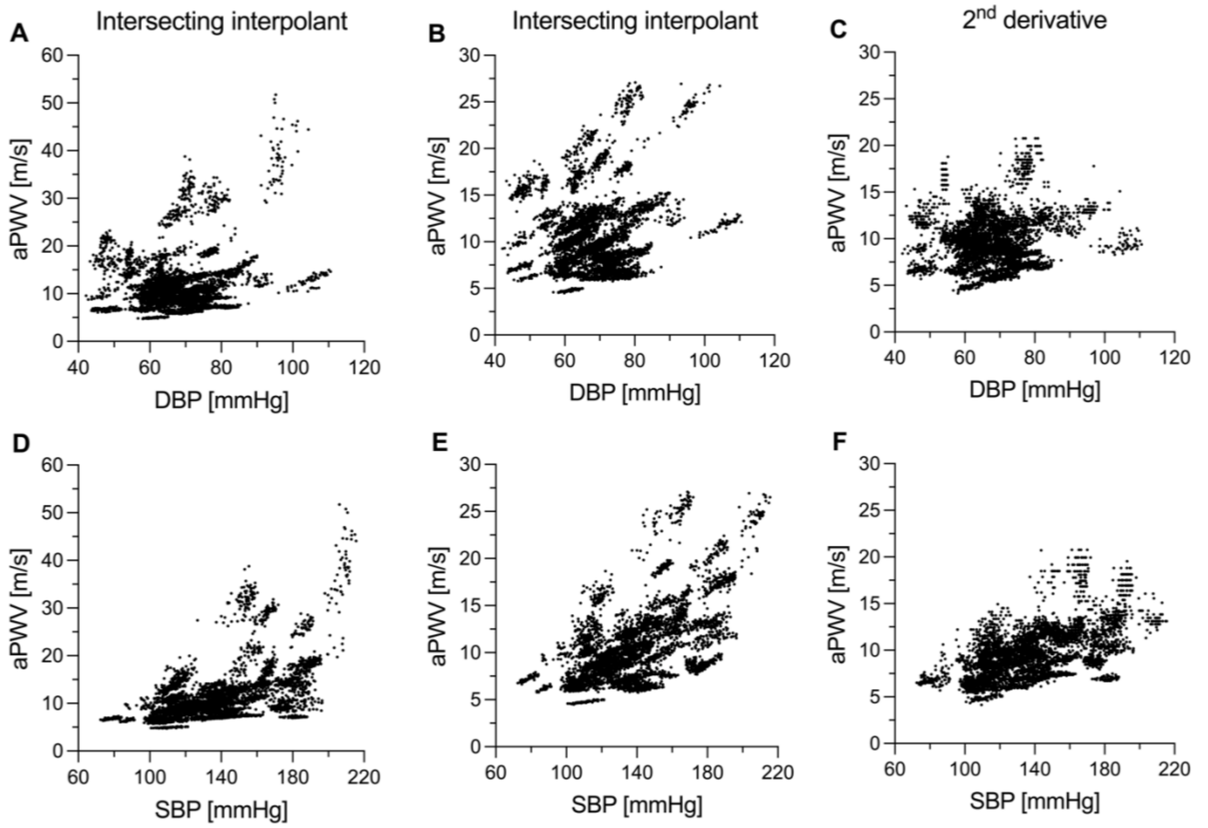

Figure S6 – Relationship between beat-to-beat changes in blood pressure and PWV for all patients of our cohort. Each datapoint represents a single heartbeat from a patient of our cohort.

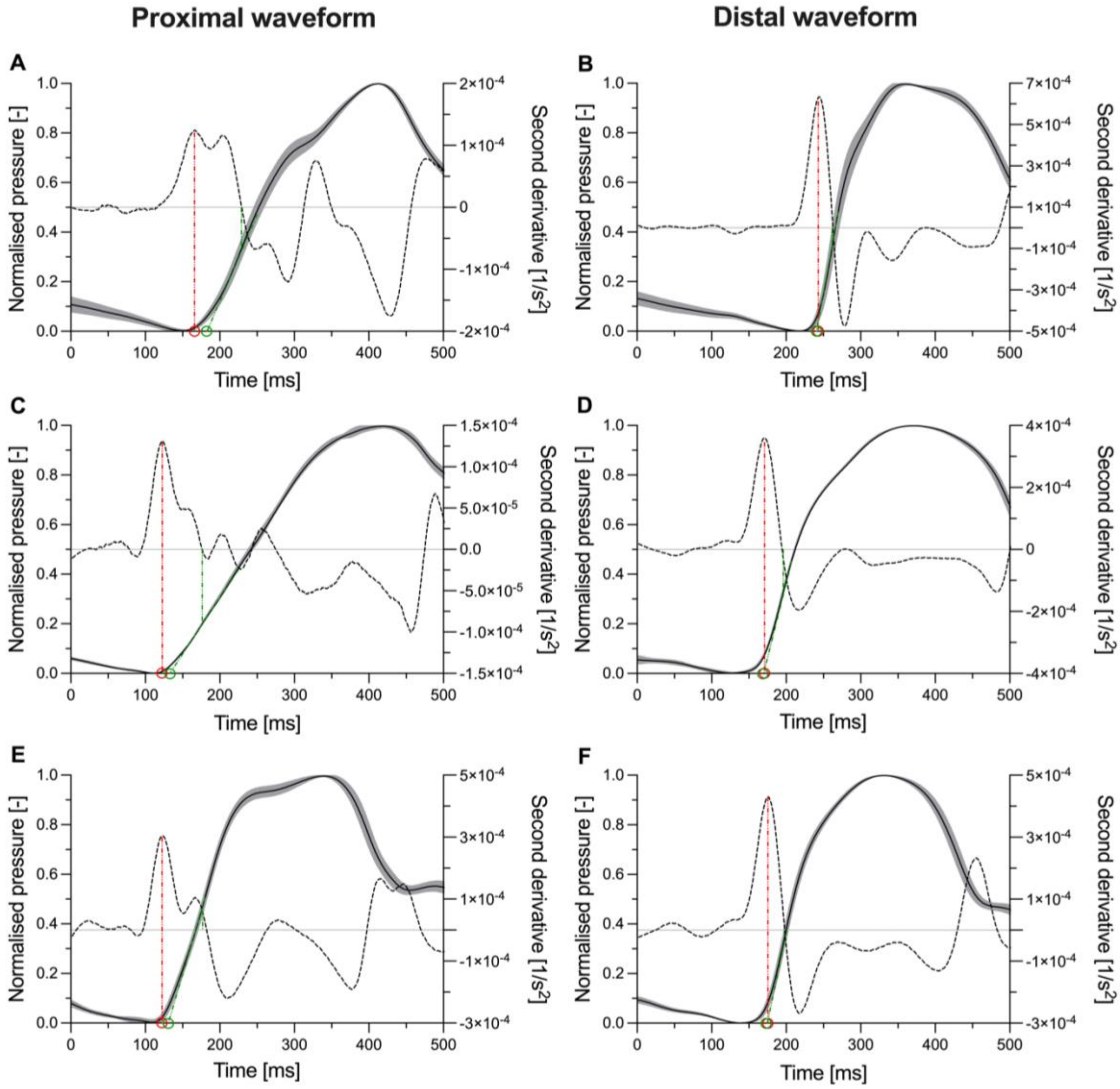

Figure S7 – Shape analysis of the ascending aortic (Panel A) and iliac bifurcation (Panel B) pressure waveforms of three patients included in this study. Each graph presents the ensemble averaged normalised pressure heartbeat of one patient (solid black line). To build these ensemble averaged curves, each heartbeat was normalised by first subtracting its diastolic pressure values and then dividing by the systolic peak. Grey areas indicate ± 1 standard deviation around the ensemble averaged curve. Dashed black lines shows the second derivatives of the ensemble averaged waveforms. Red and green lines and circles show the waveform processing for the identification of the waveform foot according to the intersecting tangent and second derivative methods, respectively. The upstroke of the iliac pressure waveform (Panels B, D and F) presents a sharper upstroke than that of the ascending aorta waveform (Panels A, C, and E), as clearly shown by the well-defined peak of its second derivative. Conversely, the second derivative of the upstroke of the proximal waveform presents a broader peak with often more than one local maxima, which complicates the identification of the systolic foot and compromises agreement between different methods. Indeed, inter-method agreement on the foot identification was much stronger at the iliac bifurcation than at the ascending aorta.
